# Exploring stakeholders’ experiences on implementing family medicine in urban South Africa

**DOI:** 10.4102/phcfm.v17i1.4675

**Published:** 2025-04-24

**Authors:** John M. Musonda, Shabir Moosa

**Affiliations:** 1 Department of Family Medicine and Primary Care, Faculty of Health Sciences, University of the Witwatersrand, Johannesburg, South Africa

**Keywords:** family physician, family doctor, private general practitioner, primary health care, universal health coverage, experiences or perceptions, South Africa

## Abstract

**Background:**

South Africa recognised family medicine as a medical speciality in 2007. The discipline plays a significant role in strengthening primary health care. However, the experiences of family physicians, private general practitioners and other stakeholders on implementing family medicine in urban South Africa remain unexplored.

**Aim:**

To explore stakeholders’ experiences in urban South Africa on implementing family medicine.

**Setting:**

All participants were from Gauteng province, South Africa.

**Methods:**

The evaluated lived experiences of implementing family medicine as a clinical discipline. A descriptive, explorative qualitative study was undertaken using semi-structured, in-depth interviews with purposively selected individuals from October 2019 to December 2020. Thematic data analysis used MAXQDA version 2020.

**Results:**

Four major themes emerged. Most participants felt a disparity between private and public primary care services, with the latter having optimistic views about health reforms. Further, private general practitioners’ clinical skills and competencies needed strengthening, and mixed staffing for primary care teams was envisioned. Many participants had ill-defined ideas about family medicine’s impact and prospects but agreed that more resources were needed to improve it.

**Conclusion:**

The study highlights the participants’ first-hand involvement in implementing family medicine. The findings may enhance knowledge of primary care services, clinical skills and staff composition. Further research is recommended.

**Contribution:**

The study provides insight into recognising family medicine, which preceded the government’s efforts to introduce Universal Health Coverage to be funded through the National Health Insurance.

## Introduction

Family physicians (FP) are based in the primary health care (PHC) setting, and their contribution to the optimal functioning of the team could enhance performance and patient outcomes.^1^ Contrary to the holistic approach, biomedical perspectives address the specific clinical needs of the individual. The former is entrenched in the tenets of primary care, which include first-contact access, continuity of care, comprehensiveness, patient coordination and community engagement. The presence of FPs in the configuration of the primary care team was associated with better clinical performance, processes and patient outcomes at the district hospital and not necessarily at the community health centre level.^1,2^

A study conducted in Johannesburg, South Africa, demonstrated that integrated PHC teams did not exist.^1^ Change management and policy reviews were required to create proper integration in primary care clinics. The findings highlighted the need for healthcare reforms in South Africa (SA).^1^ Authors proposed primary care re-organisation around FPs to attain skilled PHC teams. The findings were supported by others who reported that for African countries to attain sustainable development goals (SDGs), they needed to fast-track FPs’ training and redefine their roles to reach their full potential.^3,4^

In South Africa, FPs are trained in a 4-year postgraduate qualification, and the Health Professions Council of South Africa (HPCSA) registers them as medical specialists.^5^ Further, the public sector appoints registered FPs as medical specialists, remunerated at par with other specialists. However, some specialist family physicians (SFPs) working in the private sector are at the general practitioner (GP) level and cannot order investigations and treatments like other specialists. Hence, they are not reimbursed according to their HPCSA registration as specialists.^5^ However, the situation is changing, and advocacy efforts are articulated in a combined position statement by the South African Academy of Family Physicians (SAAFP) and the South African Society of Specialist Family Physicians (SASOSFP).^5^

In a country where about 15% of the population cannot afford private medical care, financial resources are concentrated in the private sector, and skilled health professionals tend to follow the flow of resources.^6^ With the advent of health reforms, there were fears that SA healthcare professionals could migrate abroad in anticipation of strict regulations under the National Health Insurance (NHI).^7^ The net result would be a staff shortage, inadequate GP training and a lack of a transparent financial model for health reforms. However, it was widely expected that NHI could be financed by repurposing the existing health budget and introducing tax imperatives.^8^

At the time of the study, the South African government was pushing ahead to implement the NHI, an ambitious but controversial plan to bring free quality healthcare to all citizens by pooling public and private sector resources into a single entity to procure and pay for health services. The NHI was broadly outlined in the White Paper, and the Bill was subsequently available for public comment.^6,7,8^ However, critics were alarmed by the sweeping health reforms proposed in the NHI, citing the potential for corruption, inefficiency and mismanagement of State resources.^9,10^ However, the State President signed the *NHI Act no. 20 of 2023* into law on 15 May 2024 and was gazetted the next day.^11^

This study aimed to explore the experiences of academic FPs, private GPs and key stakeholders in urban Gauteng province, South Africa, in implementing family medicine as a recognised speciality. That was particularly important because family medicine was recognised as a speciality gazetted in 2007 and before the introduction of PHC re-engineering in 2012 as a prelude to health reforms.^8,9,12^ Specific objectives were to explore participants’ direct involvement in implementing family medicine as a clinical discipline in private or public primary care, views on healthcare reforms or NHI, GP skills and competencies and PHC staffing composition.

## Research method and design

### Study design

The study applied an explorative qualitative research design, utilising in-depth interviews of academic FPs, private GPs and key stakeholders to address specific study objectives.

### Study setting

We recruited participants from Gauteng province, which has the largest population, is highly urbanised and is the country’s economic hub. The province had the highest concentration of private GPs in South Africa, with the largest share of 15.83 million or 25.1% of the South African population estimated at 63.02 million in mid-2024.^13^ The province had the highest concentration of private GPs in South Africa.^14,15^ Researchers were full-time FPs and affiliated academics with the University of the Witwatersrand Department of Family Medicine and Primary Care. Some of the participants were known to the researchers.

### Study population and sampling

The study population included experienced academic FPs, private GPs, mentors and healthcare managers from the public and private health sectors. The participant characteristics are listed in Table 1.

**TABLE 1:** Participants’ characteristics.

No.	Participant	Age (years)	Gender	Race	Qualified (year)	Experience (years)	Position
1	FP1	49	F	I	2004	16	Principal FP
2	FP2	51	M	B	1992	28	Senior FP
3	FP3	47	M	B	2005	17	Senior FP
4	FP4	65	F	B	1983	37	Principal FP
5	FP5	56	F	B	1989	31	Senior FP
6	FP6	67	M	W	1976	44	Principal FP
7	FP7	54	M	B	1992	28	Senior FP
8	FP8	47	M	B	2002	18	Principal FP
9	FPSH1	45	F	I	1992	28	PH Specialist
10	FPSH2	65	M	B	1980	40	PH executive
11	FPSH3	48	M	B	1998	22	PHC executive
12	FPSH4	48	F	B	1999	21	Deputy director
13	GP1	58	M	B	1988	32	GP part-time
14	GP2	54	M	B	1993	27	GP part-time
15	GP3	65	M	B	1980	40	Trained GP (FP)
16	GP4	58	M	B	1990	30	Trained GP (FP)
17	GP5	45	F	B	2004	16	Trained GP (FP)
18	GPSH1	62	M	B	1989	31	GP mentor†
19	GPSH2	75	M	W	1970	50	GP mentor†
20	GPSH3	78	M	W	1965	55	FP/mentor†
21	GPSH4	69	M	W	1974	46	FP/mentor†

FP, family physician; PHC, primary health care; FPSH, family physician stakeholder; GP, general practitioner; GPSH, general practitioner stakeholder; PH, Public Health; I, Indian; B, Black; W, White; F, female; M, male; No., Number.

† , Participant introduced to the researchers by the South African Medical Association (SAMA).

Purposive sampling of information-rich participants was based on seniority and experience in family medicine in the public or private health sectors. Eleven academic FPs, 10 private GPs and key stakeholders were interviewed. J.M. identified vital participants. Further, participants in the private health sector were identified and cascaded to the researcher by the South African Medical Association (SAMA), a professional organisation for members registered with HPCSA. However, the South African Medical Association Trade Union (SAMATU) was not involved in the study.

### Data collection

Researchers developed a semi-structured interview guide collaboratively and in line with the study objectives. This was piloted with two interviews and feedback, after which the interview guide underwent no further changes. The piloted interviews did not form part of the final analysis.

Participants were invited in advance by telephone call, email, text or WhatsApp message. In-depth interviews took place from October 2019 to December 2020. Fifteen (15) were interviewed in person, one-on-one, while six were virtual via Zoom or WhatsApp platforms because of the coronavirus disease 2019 (COVID-19) pandemic. Information sheets detailing the topic, aim, objectives and written informed consent were forwarded to each participant. Interviews were conducted in English by John M. Musonda (J.M.M.) and lasted between 30 min. and 45 min. No translation was needed. A digital audio recorder was used to record the interviews. The researcher listened, probed and kept reflective notes prepared. Interviews were transcribed verbatim by an independent transcriber.

### Data analysis

Systematic text condensation analysis began soon after conducting each interview to get preliminary data that would determine saturation, where a sense of closure was attained because new data yielded redundant information. Despite reaching the theoretical sufficiency or saturation point of 10, the researcher continued interviewing all targeted participants to get a deep exploration and understanding of the topic. Each transcript was saved using a pseudonym to ensure anonymity and exported to the MAXQDA version 2020 (Verbi Software, Berlin, Germany) for thematic analysis.

An evaluation process ensured trustworthiness through credibility, transferability, dependability and confirmability. The researcher independently coded the data and discussed it with an independent coder to reach a consensus on themes. The results reflected the participants’ direct involvement in implementing the speciality rather than the researcher’s values. Findings were corroborated further by interviewing beyond the saturation point and information converged.

Moreover, different qualitative data analysis steps were applied. By familiarisation, each transcript was repeatedly examined in detail and structured around codes or common ideas and sub-codes. Indexing codes from the data meant codes matched the collected data segments, highlighted and dragged to the pre-set and defined codes and sub-codes. The text was coded for content, patterns and processes. The code and retrieval systems were activated once code segments were created across all 21 documents. Codes were continually compared to each transcript to ensure similar concepts were consistent.^16,17,18,19^

By thematic indexing, final codes from related ideas were reviewed, clustered and linked to form themes. Virtual mapping of concepts reduced data and the analysed themes. Tables, graphics, code clouding and ideas were obtained by using the MAXQDA software. Charts were used to interpret data on how participants foresaw, knew, imagined or encountered the implementation of family medicine. The researcher took notes on ideas, thoughts, feelings and judgements and used them in the analysis.^18,19,20^

### Ethical considerations

Approval was sought from the University of Witwatersrand Human Research Ethics Committee (approval number: M1811115). The National Health Research Database reference number was GP_201903_02 (1). Each participant was given information and informed voluntarily, and written consent was obtained before participating in the study. The data collected were anonymous, confidential, safe and secure, with password protection. Participation was voluntary, and participants were allowed to opt-out at any stage.

## Results

Thirty-five (35) participants were invited, but only 21 were interviewed. There were various reasons for non-attendance, ranging from not responding to the call on the day, being busy at the time, ill health, retirement and failure to honour the appointment for no reason. There were 11 participants from the public health sector and 10 from the private. After 10 interviews, we did not obtain data that explained new concepts.

The mean age of participants was 57.4 years, ranging between 45 years and 78 years. Most were male (71%) and black people (71%). Work experience varied between 16 years and 55 years. Table 1 shows participants’ characteristics.

The combined parent codes, sub-codes and categories yielded four (4) major themes: Disparity in the delivery of primary health care services; Participants’ attitude to health reforms; Strengthening private general practitioners’ clinical skills and competencies and Staff composition in the primary care teams.

### Disparity in the delivery of primary health care services

#### Heavy workload and overcrowded public clinics

With the widening disparity in PHC services, most participants felt the private sector offered better benefits. Further, there was consensus on resource challenges faced by the public sector, leading to overcrowding, long patient waiting times and poor patient care experience. Many participants highlighted heavy workloads and constrained resources in the public sector as significant challenges. Delays in providing public sector services were consistently reported; some felt the solution was universal healthcare coverage:

‘But if you’re in an urban setting, like Gauteng, where you have a lot of private practitioners and public sector facilities …. yes, they may be initially an impression that you need to go towards the private health sector for better services.’ (FPSH1)

‘When you reach a facility with few trained people as health care providers, there will be a huge backlog, and people always feel the service is terrible like the booking system doesn’t work, and then say, why book, because if whether I book or I don’t book, I’ll still stay in the facility for four hours or five hours.’ (FPSH2)

#### Status of specialist family physicians in the private and public health sectors

Although most participants had perceptions of high-quality service delivery in the private health sector, the initial challenges in limiting the ordering of tests, treatments and reimbursements for FPs by medical schemes and the private health sector generally at the time worsened the disparity in the delivery of services at the PHC platforms. The participating SFPs in the private health sector reported feeling demotivated because they were reimbursed like GPs. On the contrary, the public sector appointed and remunerated SFPs at par with other specialists registered with the HPCSA, as shown below:

‘The private GP knows how to run his practice better than us. For instance, there’s a capitation system, and he needs to see a thousand patients. If the patient gets sick, they make an appointment with him. They come to his surgery, he sees them, he gives them medicine from what they stock the practice with, and he’s got a licence to hand out medicines. That is quality service.’ (FP6)

‘But in private practice, there’s no recognition of the family physician, starting from the medical schemes. So far, family medicine, physicians, and general practitioners are treated the same. There’s no big difference in payment-wise for doing procedures or the consultation, which is now demotivating for family physicians.’ (GP4)

### Participants’ attitude to health reforms

#### Improved access and equity in primary care services

Although there was consensus on the need for healthcare reforms in SA, most participants from the public health sector were more optimistic than private ones. They reported that healthcare reforms could improve healthcare provision by enhancing standards in primary health care services, particularly access to essential services and reinforcing equity regardless of the anticipated challenges as exemplified below:

‘Okay, I think NHI is okay; let me explain what I think. From one point of view, correcting the inequalities and injustice which is happening in South Africa, considering the apartheid history, we need to change that. We need to do it. But it involves a lot of money.’ (FP3)

#### Increased state control of health services

Most participants from the private health sector expressed concerns that health reforms were a way of introducing more controls through legislation for private GPs, specialists, hospitals and medical schemes. While they agreed on the principle of equity, they did not support the reforms because they felt cheated because of inadequate consultation with them. Hence, they argued that reforms were vague, not achievable, ‘a very kind of utopian’ and ‘money-driven’, as highlighted below:

‘Listen, I have listened to so many presentations about NHI, and I don’t think it will happen in my lifetime. And I don’t know whether there will be any difference for a family practice than now, except where your next paycheck comes from. The NHI will meet resistance because it’s trying to impose things, and once you try to impose something on a doctor.’ (GPSH3)

### Strengthening private general practitioners’ clinical skills and competencies

Most participants felt that acquiring clinical skills and competencies for private GPs was vital. It should be a primary requirement for them to fulfil before embarking on general practice in the community once they obtain their qualification as doctors or MB ChB and the equivalence. Also, they acknowledged the gap in acquiring training in clinical skills and the competencies needed for them to function better. For the formal private GP skills, participants suggested enrolling for a diploma in family medicine, and reinforcing formal or informal mentorships would be key.

#### Fast-tracking general practitioner skills by enrolling for a diploma

‘You know what, I’ve been in the same practice for nearly twenty years, and you must remember things were very different years ago. That’s why I decided to enrol for the diploma. I didn’t realise it beforehand, but it’s wonderful to get updates, and the way of teaching medicine has changed completely. I think everyone should do the diploma course and improve.’ (GPSH2)

#### Reinforcing informal and formal mentorships

Many participants mentioned their direct involvement in acquiring and teaching clinical skills. They reported the need to build a formal or informal relationship between an experienced or senior person and often a younger person to guide, influence or direct the latter to achieve personal and professional development, as shown below:

‘I had to learn much of the family medicine being mentored; it didn’t come through in the family medicine degree. So, get experienced family doctors that have, I suppose, mentorship and upskilling education to come and teach younger doctors, which we’re doing informally on our WhatsApp groups. And via emails and conversations.’ (GP7)

### Staff composition in the primary care teams

#### Working in more mixed primary care teams

Most participants believed a more mixed staff composition or group practice with multidisciplinary skills would be the norm in the future primary care setting. Some reported that conformity to staff service-level norms and standards was a tool for determining the necessary skills:

‘Ja, you need a family physician in the centre because you can connect to any discipline. We also need to train and empower our nursing staff. We also need support staff and WBOT [*Ward Based Outreach Teams*] teams to go to the community. If we do that, the primary health care will be strengthened. Don’t forget the public health specialists, also.’ (FP2)

‘And in our setting, I work with an entire multidisciplinary team from the dietician to a physio, OT [*Occupational Therapist*], speech therapist, nursing staff, etc. So that the entire team would be something that I would hold as vitally important from now on, with me being the head and then everyone else forming part of the prong of that team.’ (GP6)

#### Behaviours promoting community participation

Most participants felt that to improve understanding and ownership of primary care services, it was vital to involve and engage the community to generate input in the planning, designing and delivering relevant services. This may help them appreciate possible opportunities that may exist for them. Several participants still felt that teams that practised behaviours that favoured community empowerment were vital in sustaining the delivery of better PHC services, as shown below:

‘So, the community do not understand the difference between me as a GP and a family physician. Let’s talk to them, partnering, explaining and educating them about all these things to get a better outcome for their medical conditions.’ (GP5)

## Discussion

**FIGURE 1:**
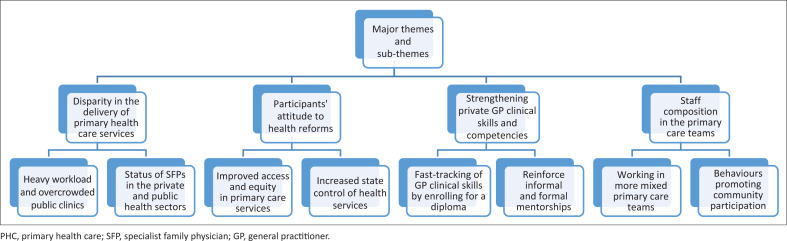
Thematic map of major themes and subthemes depicting participants’ experiences implementing family medicine since it was recognised as a speciality in 2007.

Four major themes emerged from this study. Most participants felt a disparity between private and public primary care services, with the latter being more optimistic about health reforms or the NHI. Further, most participants reported that private GPs’ clinical skills and competencies needed strengthening, and they envisioned a mixed staff composition for primary care teams (See Figure 1).

The NHI bill was signed into law, the *NHI Act no. 20 of 2023*, on 15 May 2024 and gazetted the next day.^11^ Nonetheless, the *Act* is presented in a manner that requires dialogue, further deliberations and time. The positioning of family medicine specialists under NHI needs clarification because the *Act* is silent about it.^11^ Interestingly, a prelude to the government’s efforts to introduce Universal Health Coverage financed by NHI was the PHC re-engineering over a decade ago.^6^ Also, it is 17 years since HPCSA formally recognised family medicine as a speciality.^12^

Regardless, it is important to assume that the speciality would not be side-lined because Gauteng and other provinces took a proactive step over 18 years ago to create family medicine specialist posts to strengthen PHC.^21^ The intervention aimed to improve PHC in Gauteng around FPs as clinicians, capacity builders, change agents, collaborators, community advocates and clinical leaders.^22,23^

Differences in the provision of PHC services between public and private health systems may be explained based on the inherent attributes of each system. For public health services, it is usual to find a heavy workload, as seen by long patient waiting times, overcrowding and staff constraints. Further, the clinics have drug stock-outs and no patient user fees.^5^ By contrast, private health care is considered a gateway to public service, with perceptions of high-quality, family medicine recognition challenges, interactions with medical schemes and inaccessible services by many.^6,10^

Although participants generally agreed with the overall goal of the reforms or NHI, some views were contradictory. The majority expressed concerns ranging from potential corruption, inefficiency and mismanagement of State resources. Under new reforms, corporate governance is heavily skewed towards the state-centric control of public and private healthcare. While many participants were hopeful about opportunities and the future, others mentioned negative experiences and threats to family medicine, which may have resulted from inadequate messaging from the State, in keeping with studies from Cape Town and the Eastern Cape.^9,10^

Accelerating the pace of clinical skills acquisition among private GPs is needed.^22^ This would require public and private academic institutions like the Foundation for Professional Development (FDP) and others to commit to the initiative and introduce incentives to attract public and private GPs and SFPs to participate in refresher courses and further training. Further, family medicine is beginning to be recognised at the same level as other specialities in the private health sector.^5^ Consequently, this demands a multi-pronged approach from key stakeholders, academics and the State to fast-track processes to implement family medicine fully.

A national diploma in family medicine is a good start in improving GP skills.^22,23^ Appropriateness of skills highlights the need for clinical skills to be prioritised and certified as the primary requirement to independent general practice, besides mentorship. Several avenues are proposed to enhance the training and development pipeline, including leveraging technology such as virtual contacts, online refresher and diploma courses and continuous professional development (small meetings, lectures, conferences and workshops).

The envisioned staff composition in the primary care team is mixed staff skills, which appears favourable. However, reports from the Eastern and Western Cape on the attitude of GPs to the NHI reported challenges in messaging and collaborative efforts by the State, which may impact funding the posts.^9,10^ Many participants felt the State needed to do more to engage them on NHI. They claimed to have obtained information about NHI through radio, television and social media. When people go to primary health care facilities for their needs, they expect to be served without being sent up and down from one facility to another. That implies that healthcare providers should provide various services by teamwork and possess multiple skills. People’s physical, mental and social needs could be addressed when health facilities have appropriate infrastructure and skills mix, conform to staffing norms and standards and improve collaboration between public and private health services.

Appropriate staff, skills mix, efficient multidisciplinary teams, management of essential functions, strong community participation and better recruiting and contracting practices could be considered important for better health outcomes.^6^ Importantly, innovation around the package of services and well-designed government contracts for private healthcare providers would be regarded as enabling the full implementation of health reforms with a specific focus on providing minimum acceptable essential health services to all South Africans and permanent residents.^6,7,8^

Family physicians are recognised medical specialists for registration purposes by HPCSA and employment by the State.^12^ Moreover, some respondents explained that PHC clinic staff only knew of FPs when the latter were introduced in meetings. That is important because it highlights that clinical skills alone as specialists may not position FPs better than non-specialist generalists, mainly when the former is allocated to less-resourced public clinics in urban areas, where it is challenging to perform basic medical tests and surgical procedures. Further, many participants mentioned that some FPs’ clinical skills did not appear superior and could have appeared better in public primary care settings, which may have implications for the past family medicine training programmes. However, an FP’s clinical skills and competencies include being a competent clinician or consultant, change agent, community advocate, critical thinker, collaborator and capacity builder. Besides that, the FP should be a manager of resources.^22,23^

The future of family medicine under health reforms is undefined in South Africa. However, opportunities exist through role clarity, repositioning of FPs, advocacy and recognition. Family medicine is now more recognised in the private sector than ever before. This implies that the family is taking giant strides towards full recognition by all stakeholders.^5^ Subsequently, most public respondents viewed improving accountability in the private sector as a critical factor for better health reforms. Family physicians were few and thinly scattered across Gauteng province although it has one of the highest concentrations of private GPs in South Africa.^14,23,24^

Although many participants generally perceived public services as poor, interventions were reported from both the Gauteng Provincial Government and the State to support improvements in delivering primary health care services to communities.^11,21^ The most critical aspect in strengthening PHC’s readiness for the NHI implementation would probably be the development of the management and leadership capacity of numerous front-line managers. This is a crucial ingredient for the success of the health reforms in South Africa.^25^

The World Health Organization (WHO) reviewed relevant documents for granular data regarding the decentralisation and implementation of Universal Health Coverage (UHC) from NHI pilot sites in the country’s health districts from September 2014 to December 2015.^26^ The authors reported practical issues, ideological conflicts and positive discrimination for the richer or healthier individuals.^27^ However, specific areas needed for improvement in achieving sufficient UHC coverage for South Africa would include the administration and management of health institutions and district management capacity at all levels, particularly in rural areas. The finding supports the assertion about management and leadership made by the WHO.^25^

### Strengths and limitations

The study has some strengths. The findings add value to the knowledge and understanding of implementing family medicine as a speciality in an urban area and the emergence of family medicine in delivering private and public primary care in the country. Further, the study highlights the need to improve the acquisition of GP skills and competencies and to attain a more mixed staff composition for primary care teams.

A primary limitation is the sample, which depended on the availability of the selected individuals. By limiting our sample to eight academic FPs who were in the public sector, five FPs in the private, three private GPs and two who had part-time contracts with the State and their stakeholders, we could not learn from GPs who were purely from the public sector because we did not target them and may have led to potential selection and participation bias. Because the researcher selected whoever was easily accessible or had specific characteristics, the study sample may not adequately represent the population. Hence, the findings may not be generalised to other similar geographical areas. Other potential biases that could be introduced include researcher, confirmation, social desirability and interpretive biases.

Although SAMA is a professional body of members registered with HPCSA, its involvement in recommending that some participants be interviewed may be considered selection bias. To mitigate selection bias, the research question and objectives were clear and precise, and several sources were used to recruit adequate and diverse participants. Reflexivity and triangulation kept the researcher’s role and influence in balance.

The interviewer was a specialist FP known to some participants, who may have been pressured to share positive experiences rather than negative ones. Most participants expressed their emotions and language well despite the researcher not personally knowing them prior. To mitigate potential biases, the researchers attempted to be aware of the possible existence of such biases and kept interview notes. Further, the researcher used an independent person or supervisor to check findings and interpretations.

In introspection (reflexivity), researchers were full-time FPs employed in the public health system and affiliated academics at a local University. During interviews, the researcher took notes on their ideas, thoughts, feelings and judgements and examined how that could have affected the research. Some participants were personally known to the researchers, which could have influenced their responses. However, participants had no reason to withhold their experiences because the study’s benefits were explained to them. Also, there was anonymity, and the data were to be coded.

### Recommendations

The study provides an understanding of the implementation of family medicine as a discipline in urban South Africa. Providing appropriate resources would be vital in supporting the implementation of family medicine and reforms. Among others, human resource investment is critical despite available challenges.^28^ The country deliberately chose PHC as the driver of healthcare delivery. Hence, strengthening primary health services by creating FP posts to bring specific skills and competencies to primary care clinics may improve access to services, equity and better clinical outcomes.^3,4,28^

## Conclusion

The study provides insights into the disparity between public and private primary care services, the more optimistic public sector on health reforms, the strengthening of private GP skills and the more mixed staff composition envisioned for future primary care. Further, we reported on the mixed impact of FPs on public and private primary care and the undefined or misunderstood roles of family medicine specialists. The findings of this study are important in improving GP training and policy on health reforms. Future research involving the PHC staff is recommended.
